# DNA Scission by Non‐Histidine Amino‐Terminal Cu(II) and Ni(II) Binding‐Like Peptides

**DOI:** 10.1002/cbic.70397

**Published:** 2026-05-25

**Authors:** Lena K. Müller, Hanna Zhdanova, Ivan N. Unksov, Alexander Gräwe, Joshua Stahl, Viktor Stein, Daniel Tietze, Alesia A. Tietze

**Affiliations:** ^1^ Clemens‐Schöpf Institute for Organic Chemistry and Biochemistry Darmstadt University of Technology Darmstadt Germany; ^2^ Department of Chemistry and Molecular Biology Wallenberg Centre for Molecular and Translational Medicine University of Gothenburg Gothenburg Sweden; ^3^ Department of Biology Darmstadt University of Technology Darmstadt Germany

**Keywords:** amino‐terminal Cu(II) and Ni(II) binding (ATCUN) peptides, copper coordination, DNA scission, metallopeptides, plasmids

## Abstract

Metallopeptides are promising candidates for drug design, ranging from anticancer agents to antimicrobials. We report the ability of non‐His‐containing metallopeptides to induce scission of plasmid DNAs that carry antibiotic resistance genes. The peptide Ac‐Dap‐β‐Ala‐His‐PEG_4_, featuring an amino‐terminal Cu(II) and Ni(II) binding (ATCUN) motif, along with two mutants where His is replaced by Ala and Asp, form complexes with Cu(II) and Ni(II) in a pH‐dependent manner, attributed to metal coordination. The His‐ and Asp‐containing peptides demonstrate specificity for AT‐rich DNA regions. Previously, DNA scission was exclusively associated with His‐containing ATCUN peptides of other molecular structures.

## Graphical abstract

Non‐His amino‐terminal Cu(II) and Ni(II) binding (ATCUN)‐like peptides containing Ala and Asp instead of His form Cu(II)/Ni(II) complexes and induce oxidative plasmid DNA scission. The metallopeptides bind preferentially to AT‐rich DNA regions and promote nicking and linearization in the presence of Cu(II) and ascorbate. These findings demonstrate that His is not essential for ATCUN‐mediated DNA cleavage, expanding the scope of metallopeptide design for biomedical applications.

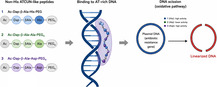

## 1 Introduction

The amino‐terminal Cu(II) and Ni(II) binding motif (ATCUN motif) is found in certain albumins such as HAS [1], BSA [2], RSA, and other structures involving histatins [3] and hepcidin [4]. The binding site of HSA, namely Asp‐Gly‐His, facilitates copper transportation within the blood. Extensive studies around the motif, the simplest being Gly‐Gly‐His, have been performed since the first discovery in 1948 [2] involving structural studies on complex formation [5], design of metal sensors [6] and also the ability to linearize dsDNA [7, 8]. Intensive studies over decades revealed a 4N coordination of the Ni(II) and Cu(II) complexes in a square planar geometry through the free N‐terminal amino nitrogen, two intervening amide nitrogen of the peptide backbone and the imidazole nitrogen of the His residue [3, 7]. During the last two decades, the ability of metallopeptides to bind and interact with DNA became a topic of great interest because of their enormous therapeutic potential. For example, compounds from the bleomycin pool, a group of glycopeptides, are used for cancer treatment because of the DNA scission ability of the metallobleomycin [9]. In the early 1990s, the binding behavior between pyrrole and imidazole‐containing polyamides was investigated, demonstrating the recognition of AT‐rich structures in the minor groove of double‐stranded DNA (dsDNA) [10]. Because of the mandatory position of His within the ATCUN motif, the presence of an imidazole moiety is guaranteed, making them scaffolds for peptides with DNA recognition abilities. Long and coworkers investigated the importance of the stereochemistry of the ATCUN motif with regard to minor‐groove binding using a dodecanucleotide scaffold [4]. Metallopeptides coordinating copper and/or nickel ions can be used for oxidative cleavage through C‐4^′^H abstraction from the minor groove [11]. Binding was investigated by Huang and coworkers and explained through hydrogen bonding of the His N_3_ imidazole hydrogen to the N_3_ of adenine or O_2_ of thymine [12]. For DNA cleavage by metallopeptides of the ATCUN motif, the oxidative pathway is described. Jin and coworkers made use of ascorbate (Asc) or KHSO_5_ in buffer systems of pH 7.4 and 7.5 for efficient DNA cleavage using Cu(II)‐metallopeptides [11]. Furthermore, substitution of Gly in the ATCUN motif to β‐Ala [13], incorporation of Lys [13, 14] and recently N‐terminal heteroaromatics [15] in the ATCUN peptides were shown to facilitate Cu redox cycling and resulting DNA cleavage. Substitution of the Gly to L‐Arg was shown to produce peptides for which AT‐specific binding is enhanced [4]. In recent years, interest has arisen in utilization of metallopeptides in diagnostic aspects. Cowan and coworkers investigated numerous applications ranging from antimicrobial nuclease and ribonuclease activity [16] over targeting the HIV‐1 Rev response element [17] to the inactivation of the angiotensin converting enzyme [18]. All those use the ATCUN‐motif and other metallopeptides. Another publication targets the West Nile virus and shows the cruciality of metallopeptides in the medicinal field [19]. While these experiments all involve coupling of the ATCUN‐motif to a selective moiety, we wanted to investigate if there is a structural preference of the motif itself aside from the AT‐rich regions that were discovered from studies with short DNA sequences. Further, we were interested in the “mandatory” His residue. Kritzer and coworkers described Cu(II)/Ni(II) binding and redox properties of substituted ATCUN motifs lacking the His moiety, coming to the conclusion that peptides without the imidazole moiety are also forming ATCUN‐like complexes with Cu(II) and Ni(II) ions, however, their catalytic activity varies [20].

## 2 Materials and Methods

### 2.1 Solid Phase Peptide Synthesis and Peptides Characterization

Ac‐DAP‐β‐Ala‐His‐mPEG_**4**_ (**1**), Ac‐DAP‐β‐Ala‐Ala‐mPEG_**4**_ (**2**) and Ac‐DAP‐β‐Ala‐Asp‐mPEG_**4**_ (**3**), and GGH were synthesized following the standard Fmoc‐SPPS using chlorotrityl chloride resin (0.966 mmol/g). After mPEG_**4**_ coupling, the resin was capped with 8.5:1:0.5 dichlormethane (DCM)/methanol/N‐ethyl‐N‐(propane‐2‐yl)propane‐2‐amine (DIEA), followed by coupling of Fmoc‐His(Trt)‐OH, Fmoc‐β‐Ala‐OH, and Fmoc‐Dap(Boc) correspondingly. Deprotection was obtained by 20% piperidine in N, N‐dimethylformamide (DMF), and the coupling efficiency was monitored by UV–vis spectra at 301 nm. All peptides were acetylated at the N‐terminus with acetic anhydride. The final cleavage from the resin was carried out in 2/2/48/48 triisopropylsilane (TIPS)/water/trifluoroacetic acid (TFA)/DCM and stirred for 1 h at room temperature. RP‐HPLC purification was performed on a C18 column (MultoKrom 100–5. 250 x 20 mm, 100 Å pore diameter, 5.0 µm particle size) following an isocratic method of 0% eluent B for 12 min followed by a linear gradient to 30% eluent B within a total of 60 min (eluent A is water (0.1% TFA) and eluent B is acetonitrile (0.1% TFA)). The HPLC system used was Waters 600 equipped with a Waters 2996 photodiode array detector. The molecular mass was confirmed by ESI‐MS (**1**: m/z: [M + H]^**+**^ calculated for C_25_H_43_N_7_O_10_ 602.31, found 602.31; **2**: m/z: [M + H]^**+**^ calculated for C_22_H_41_N_5_O_10_ 536.26, found 536.29, and **3**: m/z: [M + H]^**+**^ calculated for C_23_H_41_N_5_O_15_ 580.28, found 580.28). Collected fractions were combined, freeze‐dried and stored at –28°C. Purity of the collected fractions were confirmed by analytical RP‐HPLC on a Waters 2695 alliance system employing a Waters 2998 photodiode array detector equipped with a MultoHigh 100 RP18 column (125 × 4 mm, 100 Å pore diameter, 3.0 µm particle size, CS Chromatography Service GmbH). The gradient elution system was 0.1% trifluoracetic acid (TFA) in water (eluent A) and 0.1% TFA in acetonitrile (eluent B). The peptides 1–3 were eluted using a isocratic method of 0% eluent B for 4 min followed by a linear gradient to 30% eluent B in 20 min total run time. For the GGH‐peptide, a gradient of 0 to 15% eluent B was used. Chromatograms were extracted at 214 nm*.* The molecular weight of the purified peptides, as well as complexation of peptide 1–3 with Cu(II) (CuSO_4_, in water), was confirmed by ESI mass spectrometry on a TOF‐Q impact II spectrometer (Bruker Daltonik GmbH, Bremen, Germany) and calibrated using Bruker’s ESI‐Tune‐Mix.

### 2.2 Determination of Peptide Content Via NMR Spectroscopy

All peptide concentrations were determined by RP‐HPLC using a calibration curve with a peptide solution quantified through NMR experiments. As an external standard, N‐acetyl alanine was used. The 1H integrals of the protons within the acetyl group of N‐acetyl alanine were compared to the acetyl group at the N‐terminus of peptides **1** to **3**. The sample (water + 10% D_2_O) to be measured was immediately used as the calibration standard for the RP‐HPLC.

### 2.3 UV/Vis Spectrophotometric Titration

UV/vis titration experiment, were conducted on a Tidas MCS UV/NIR and evaluated using the Panorama program.

For pH titration studies, peptides 2 and 3 were dissolved to yield a 2 mM concentration. One molar equivalent of CuSO_4_ solution (320 mM, in water) is added and the pH lowered to < 3 with 100 mM HCl stock solution (Sigma–Aldrich). After each change in pH, a spectrum is recorded. The pH is increased steadily using a 100 mM NaOH solution (Sigma Aldrich) until pH 11. The pH titration with Ni(II) (NiSO_4_) was performed accordingly.

For Cu(II) and Ni(II) titration experiments, peptides 2 and 3 were dissolved in buffer to yield 2 mM solutions. A CuSO_4_ (16 mM, in water) solution was added in 0.1 equivalents in steps to a minimum of 1.4 equivalents. After each addition, a spectrum was recorded, and the pH was controlled. Peptides 1 and 3 are measured in Tris buffer (100 mM, pH 8) and peptide 2 in Tris buffer (100 mM, pH 10.55).

It should be noted that Tris forms complexes with Cu(II) and, less strongly, with Ni(II) [21]. However, Tris buffers have been previously used in the studies on ATCUN peptides [8, 14, 22]. Cu(II) binding and DNA cleavage were shown by several ATCUN peptides in Tris‐HCl [8]. Additionally, unbound Cu(II) precipitates as Cu(II) hydroxide at corresponding molar ratios and elevated pH [21], conditions that were avoided in our study.

For experiments on Cu(II) release, CuSO_4_ and glutathione (oxidized, Carl Roth) were dissolved at 100 and 75 mM, respectively, in Milli‐Q ultrapure water and added to the larger volume of the peptide **1** **–** **3** solutions in the Tris buffer.

### 2.4 Plasmids

pUC19 was purchased from ThermoFisher Scientific, pACYCT2 was gifted by Luc Ponchon [23] (Addgene plasmid # 45 799), and pASK‐IBA3plus was purchased from IBA BioTAGnology, cat. no. : 2‐1402−000.

All plasmids were transformed into DH10β competent cells and amplified. The plasmids were further isolated and purified using the NucleoSpin Plasmid DNA purification kit by Macherey–Nagel. Maps of plasmids were generated using SnapGene.

### 2.5 DNA Scission in Solution

All solutions were prepared in 25 mM Tris buffer, pH 7.4, following referred to as the buffer. Peptides **1**–**3** were dissolved in the buffer to a concentration of 100 µM. Sodium ascorbate was added to a final concentration of 100 µM. Copper ions (CuCl_2,_ Sigma–Aldrich) were added in a deficient amount to guarantee complexation; the final concentration was 90 µM. pUC19 plasmid DNA was used in different concentrations. For scission experiments in solution without further gel extraction, around 500 ng of DNA were used. For gel extraction experiments, 1 µg of DNA were used. For the positive control, BamHI‐HF was used to cut and linearize pUC19. To evaluate the needed volume of DNA, the concentration was measured using a NanoDrop ND‐1000 spectrophotometer (Thermo Scientific, USA).

### 2.6 Gel Extraction and Purification

1% agarose gels in TAE buffer were run at 100 V for 120 min and the linearized bands were cut out. The DNA was extracted using the NucleoSpin Gel and PCR Clean‐up (Mackerey–Nagel). Concentration of DNA was measured using the NanoDrop at 260 nm.

### 2.7 PCR

Pipetting schedule was followed according to the Table 1 and PCR cycles protocol is summarized in Table 2. Tables 3, 4 and 5 represent the lists of the primers which were used for pUC19, pASK‐IBA3, and pACYCT2 plasmids, respectively.

**TABLE 1:** Pipetting schedule PCR.

Name	Sample	Primer (fw, rv)	dNTPs	Buffer	Water	Phusion	DMSO^a^
Volume	1 µL	2.5 µL	1µL	10 µL	33 µL	0.4 µL	1 µL

a when DMSO is added, the volume of water decreases by 1 µL.

PCR buffer: 5X for Phusion from New England Biolabs.

**TABLE 2:** PCR cycling.

Step	Temp,°C	Time	Circles
Initial denaturation	98	2 min	1×
Denaturation template	98	20 s	35×
Annealing	54/ (56)	20 s	35×
Extension	72	40 s / (90 s)	35×
Final extension	72	3 min	1×

**TABLE 3:** Primers pUC19.

Area	Name	Sequence	Binding site
A	pUC A fw	TCTCATGACCAAAATCCCTTAACGTGAG	2237 .. 2264
A	pUC A rev	GAGCGGTATCAGCTCACTCAAGG	324 .. 347
B	pUC B fw	CTTTCCTGCGTTATCCCCTGATTCTG	280 .. 305
B	pUC B rev	GGTCTGACGCTCAGTGGAACG	2269 .. 2289
C	pUC C fw	CCACTCGTGCACCCAAVTGATC	1389 .. 1410
C	pUC B rev	TATCCCCTGATTCTGTGGATAACCG	291 .. 315

**TABLE 4:** Primers for pASK‐IBA3.

Area	Name	Sequence	Binding site
A	pASK A rev	CCACTCGTGCACCCAACTGATC	1051 .. 1072
A	pASK A fw	TTTGTCTGCCGTTTACCGCTAC	321 .. 342
B	pASK B fw	TCTCATGACCAAAATCCCTTAACGTGAG	2514 .. 2541
B	pASK B rev	GCTTAATGCGCCGCTACAGG	365 .. 384
C	pASK C fw	GGTGCCTCACTGATTAAGCATTGG	1780 .. 1803
C	pASK C rev	GGTCTGACGCTCAGTGGAACG	2546 .. 2566
D	pASK D fw	AACGCTGGTGAAAGTAAAAGATGCTG	1023 .. 1048
D	pASK D rev	CACATTTAAGTTGTTTTTCTAATCCGCAGATGATCAATTCAAGGC	2380 .. 2425

**TABLE 5:** Primers for pACYCT2.

Area	Name	Sequence	Binding site
A	pACY A fw	TGTCTCATTCCACGCCTGACACTC	2185 .. 2209
A	pACY A rev	CCGGAAGCTTCTCATTAGGCACCGGGATCTCGA	2702 .. 2724
B	pACY B rev	TCAAATGTAGCACCTGAAGTCAGCC	2587 .. 2611
B	pACY B fw	GGGCTTGTTAGCAGCCGGATCTCACAGCGGTTTCTTTACCAGACTCGAG	322 .. 346
C	pACY C rev	GTGTCAGGCGTGGAATGAGACAAAC	2157 .. 2181
C	pACY C fw	AGATCTCAATGGATATCGGCCGG	273 .. 296

### 2.8 Fluorescence Displacement Titrations

Ethidium bromide was purchased from Sigma–Aldrich, and Hoechst 33 258 was purchased from TCI. The final DNA concentration in complexes with the dyes was 1.2 μM phosphates. DNA‐ethidium bromide complexes are made with the dye/DNA phosphates ratio at 0.13, at which the binding sites are subsaturated [24]. For DNA‐Hoechst 33 258 complexes, the dye/DNA phosphates ratio was 0.015, at which the specific binding mode with strong fluorescence is manifested [25]. The DNA‐dye solutions were kept overnight or for a few days at 4°C before adding metallopeptides.

Peptides were dissolved in Milli‐Q ultrapure water to a concentration of 10 mM. All further solutions were prepared in 25 mM Tris buffer, pH 7.4. Copper ions (CuCl_2,_ Sigma–Aldrich) were added to the peptides in a deficient amount, as in the scission experiments, the concentrations of a peptide and of Cu(II) in the metallopeptide stock were 1 and 0.9 mM, respectively. As a reference for displacement by Cu(II) alone, a similar solution without peptides was made. The stock was added to the DNA‐dye solutions.

Fluorescence was recorded using a photoluminescence spectrometer FLS 1000 (Edinburgh Instruments, UK). For DNA‐Hoechst complexes, fluorescence was recorded 20 min after adding each volume of metallopeptide solution when no further changes in the spectrum were observed. For DNA‐ethidium bromide, no spectral changes were observed from the moment of metallopeptide addition.

## 3 Results and Discussion

We sought to study the functional consequences of the replacement of the His moiety on Cu(II) and Ni(II) binding, and especially DNA scission.

**FIGURE 1:**
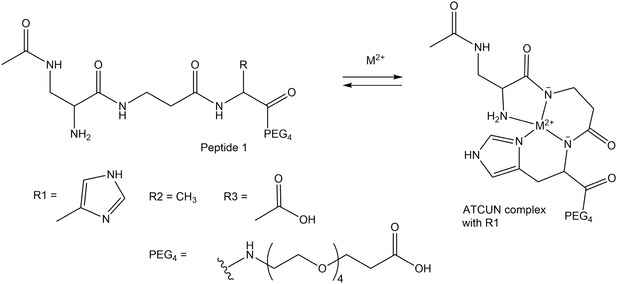
Structures of peptide 1 (Ac‐Dap‐β‐Ala‐His‐PEG_4_), 2 (Ac‐Dap‐β‐Ala–Ala‐PEG_4_), and 3 (Ac‐Dap‐β‐Ala‐Asp‐PEG_4_), as well as complex formation with a bivalent heavy metal ion M(II).

For that, we designed the following ATCUN peptide comprising the sequence Ac‐Dap‐β‐Ala‐His‐PEG_4_ (**1**) and two mutants derived thereof, Ac‐Dap‐β‐Ala‐Ala‐PEG_4_ (**2**), and Ac‐Dap‐β‐Ala‐Asp‐PEG_4_ (**3**), respectively (Figure 1). The motif is based on an optimized scaffold introduced by Imperiali and coworkers employing Dap and β‐Ala, which significantly improved Cu(II) binding and selectivity [26]. Further, the second amine functional group of Dap would allow the introduction of a fluorescence label as part of potential cell or in vivo studies, which are planned at a later time in the project and are not discussed herein. For the same reason, we have already introduce a mini‐PEG unit (PEG_4_), which is meant to increase solubility and bioavailability and prolongs potential blood circulation.

### 3.1 Metallopeptide Complex Formation

**FIGURE 2:**
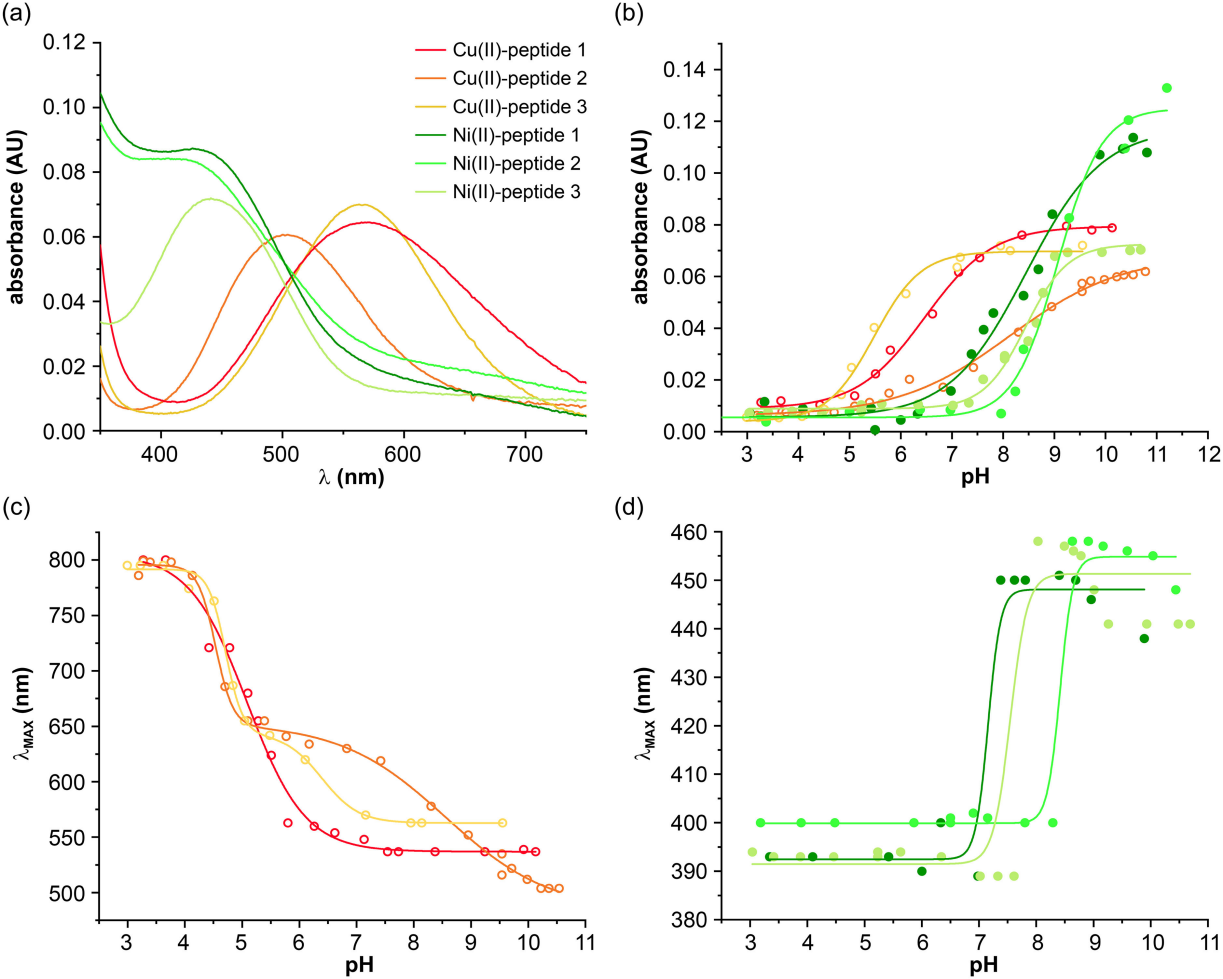
UV–vis spectra of metallopeptides (a) UV–vis spectra of peptides 1–3 upon addition of one molar equivalent of Cu(II) or one molar equivalent of Ni(II) (100 mM Tris buffer, peptide 1, 3 pH 8 and peptide 2 pH 10.55). (b) pH titration of peptide 1 (1 mM in water) and peptides 2–3 (2 mM in water) in the presence of one equivalent of Cu(II) or Ni(II). (c, d) Wavelength of the absorption maximum versus pH of peptide 1–3 in the presence of one equivalent of (c) Cu(II) and (d) Ni(II) (data for His‐peptide 1 taken from Ref. [27]).

After synthesis of the peptides and characterization by RP‐HPLC and mass spectrometry (Figures S1–S4**,** additional data for peptide 1 see Ref. [27]), mass spectrometry and UV–vis spectroscopy indicate the formation of Cu(II)‐peptide and Ni(II)‐peptide complexes for all three peptides, respectively (Figure 2a, Figures S5–S9, additional data for peptide 1 see Ref. [27]). Interestingly, during Cu(II) addition, a shift of the maximum of the characteristic metal binding absorption band was observed at values of around 1 eq. of added Cu(II) (Figures S10–S15). Further, the linear increase of the extinction coefficient upon Cu(II) addition also follows a somewhat different slope when exceeding 1 eq. of Cu(II), indicating a secondary metal binding site. Unfortunately, precipitation of metal salts at higher equivalents prevented a full characterization of that secondary metal binding site. While peptides 2 and 3 followed the same trend upon Ni(II) binding, although without any shift in their characteristic metal absorption band, the absorbance for peptide 1 reached a plateau at 0.8 eq. of Ni(II), which indicates binding of a single Ni(II) ion (Figures S13–S15).

Additionally, pH‐dependent Cu(II)/Ni(II) binding was investigated (Figure 2b). For Ac‐Dap‐β‐Ala‐His‐PEG_4_ (**1**), the absorption peak *λ*_max_ shifts from around 800 to 561 nm when pH increases from 5 to 8, which indicates the formation of the square‐planar Cu(II)‐ATCUN complex (Figure 2b,c) [5, 20, 28, 29]. Similarly, the formation of the Cu(II)‐ATCUN complex of Ac‐Dap‐β‐Ala‐Asp‐PEG_4_ (**3**) shows a discrete two‐state transition from the Cu(II) aqua complex at low pH, with *λ*_max_ at around 880 nm, to the ATCUN–like complex at high pH, with *λ*_max_ at 584 nm (Figure 2b,c). However, it is occurring in a much smaller pH range (4.5 to 6, Figure 2b) compared to the His‐containing peptide **1**. In contrast, the pH dependent Cu(II) complex formation of the peptide Ac‐Dap‐β‐Ala‐Ala‐PEG_4_ (**2**), which contains Ala instead of His, shows a different behavior, unveiling two different absorption maxima (Figure 2b,c). Apart from the Cu(II) aqua complex with *λ*_max_ at around 800 nm, *λ*_max_ at 625 nm is observed from pH 5–7.5, shifting to 492 nm when the pH is further increased to pH 10.5.

While the Cu(II) complex formation of **1** and **3** is clearly indicative of an ATCUN‐like square planar complex, employing the His‐imidazole (for **1**) and Asp‐carboxyl (for **3**) group as a ligand [5], the Ala‐containing peptide **2** must use an additional backbone amide as a fourth ligand. Most likely, the pH‐dependent shift of the absorption maximum from 625 to 492 nm reflects the deprotonation of that additional backbone amide ligand [30]. Therefore, the progression of the UV–vis titration curves of peptides 2 and 3 differs significantly.

At this point, it shall be noted that Tris buffer also forms Cu(II) complexes under similar conditions, and the resulting spectra need to be carefully analyzed [21]. However, comparing the UV spectra of the peptides with those of Cu(II)‐Tris complexes reveals distinct differences in their absorbance maxima, which led us to the conclusion that Tris does not interfere with the Cu(II)‐peptide complexes [21].

In contrast to the Cu(II) binding, binding to Ni(II) occurs in a single transition at more basic pH ranging between pH 7 and 9 for all three peptides (Figure 2b,d). It is indicated by characteristic *λ*_max_ at 438, 455, and 440 nm for peptides **1**, **2**, and **3**, respectively, which are in line with literature values of ATCUN‐Ni(II) complexes with *λ*_max_ between 425 and 450 nm [20, 29].

To probe the behavior of the peptide—Cu(II) complexes in the presence of thiols that are present in the cytosol and have been shown to cause dissociation of Cu(II) bound to ATCUN‐containing amyloid‐β peptides [31], we added glutathione at a millimolar concentration to peptide— Cu(II) solutions. Being in excess (1.5 molar equivalents) to the peptides 1–3, glutathione released most of the Cu(II) bound to Asp‐containing peptide 3, but dissociation was less complete for peptides 1 and 2 (Figure S16). This indicates that these metallopeptides can reach cellular DNA, although the thiol activity must be considered.

### 3.2 DNA Scission Experiments

**FIGURE 3:**
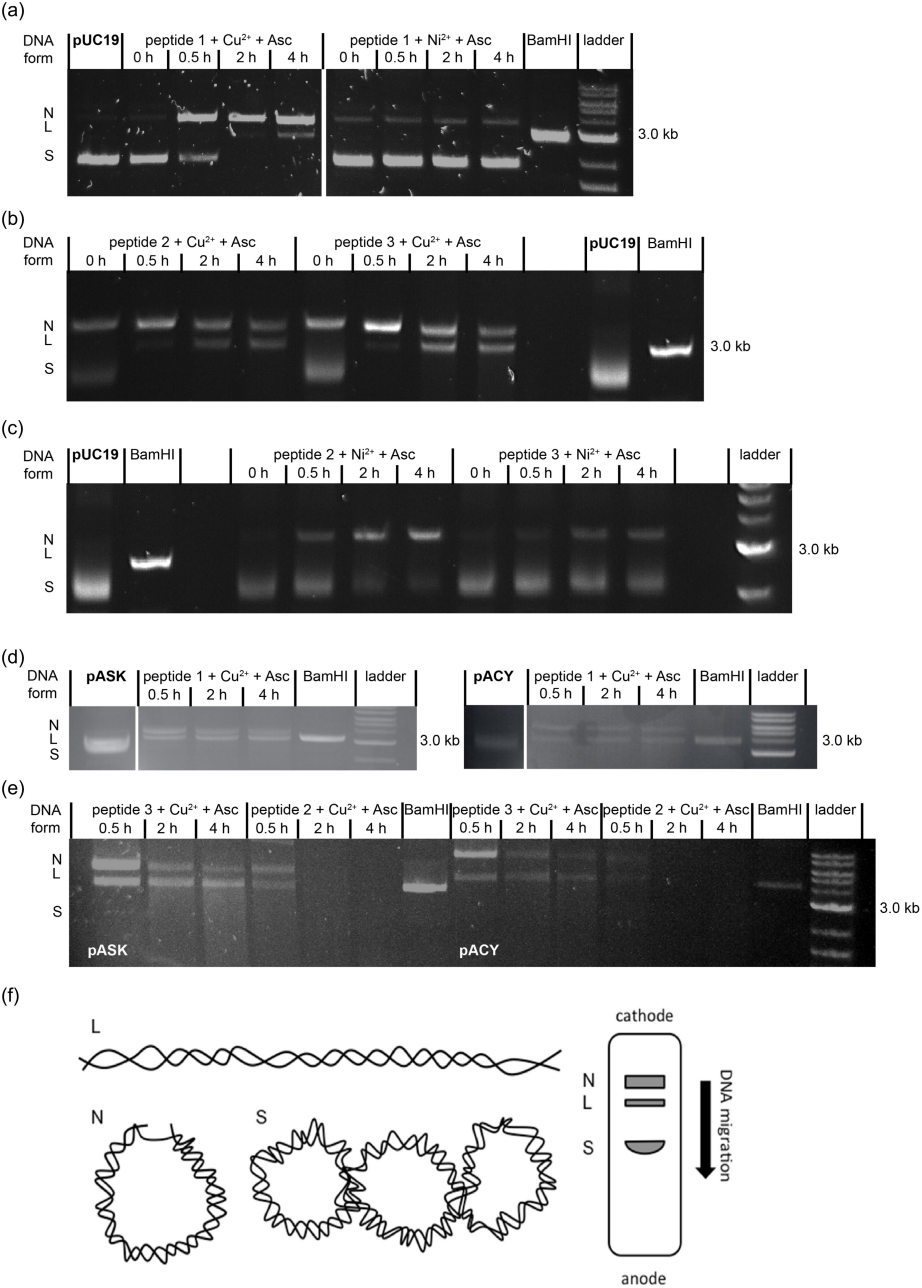
Metallopeptide‐induced DNA scission. (a–c) Scission of pUC19 plasmid (500 ng) using peptides 1–3 (100 µM) + Cu(II) (90 µM) or Ni(II) (90 µM) + ascorbate (Asc, 100 µM) within 4 h in 25 mM Tris buffer, respectively. BamHI HF is used as a positive control. Note that gel plots (b,c) are identical gel runs and were cut apart for illustration purposes only. (d,e) Scission of pASK (500 ng) and pACY (500 ng) plasmid by peptides 1–3 (100 µM) + Cu(II) (90 µM) + Asc (100 µM) within 4 h in 25 mM Tris buffer. BamHI HF was used for positive control (the original gel plots are shown in the Figures S17–S20). (f) Linearization states of dsDNA, L: linear, N: nicked, and S: supercoiled form and their movement across an agarose gel.

DNA scission by the ATCUN‐like peptides was investigated using several DNA plasmids, each possessing a different antibiotic resistance. The plasmids pUC19, used in several other studies [5, 11, 32], and pASK possess ampicillin resistance (AmpR), whereas pACY possesses chloramphenicol resistance (CmR). So far, the ability of ATCUN‐like peptides to promote DNA linearization is supposed to be based on Cu(II) catalyzed oxidation of Asc, where reactive oxygen species (ROS) are generated as described in the early 1960s by Zimmerman and coworkers [33]. The process of DNA linearization itself consists of three subsequent steps in which both DNA strands from a dsDNA plasmid (closed circular form) need to be broken [33]. Breaking the first strand leads to a nicked form, whereas breaking the second strand in proximity (around 10 bp) to the first cut results in linearized dsDNA (Figure 3f) [34].

As the cleavage of pUC19 by GGH‐ and KGGH‐ATCUN‐like peptides has been described previously [14], we study our ATCUN‐derived His‐ and non‐His‐containing peptides on their ability to linearize three different plasmids, namely pUC19, pASK, and pACY.

DNA scission experiments were followed by agarose gel electrophoresis, which allows for identifying the relevant DNA forms. In the first step, the optimal peptide concentration was determined and used for all following experiments (Figure 3f). Scission results for His‐containing peptide **1** at the concentration 125 µM do not visibly differ from those for 100 µM, but the band of the nicked DNA form slowly vanishes after 4 h at 150 µM of the peptide (data not shown). This indicates that the DNA is further cut into smaller pieces, which are too low in concentration to be visible on the gel. Thus, we further used a 100 µM peptide concentration and employed the restriction enzyme BamHI as a positive control.

All three Cu(II)‐peptides show significant scission of all three plasmids, resulting in nicked and linearized DNA (Figure 3a,b,d,e) within 4 h. In contrast, none of the Ni(II)‐peptides induce any significant amount of linearized DNA within 4 h employing the pUC19 plasmid (Figure 3a,c). Thus, the other plasmids were not further tested against the Ni(II)‐peptides.

Upon adding the Cu(II)‐peptides to pUC19, a large portion of nicked DNA is formed within 30 min, while linearized DNA becomes visible after 2 h. Further, the native supercoiled plasmid is fully consumed after this time. However, the linearization of pUC19 appears to be somewhat faster in the presence of the non‐His‐containing Cu(II)‐peptides **2** and **3** (Figure 1) compared to the His‐containing Cu(II)‐peptide **1** (Figure 1). A similar observation was made for the other two plasmids, pASK and pACY. Both plasmids are fully consumed by all three Cu(II)‐peptides within 30 min forming nicked and linearized DNA (Figure 3d,e). Interestingly, the DNA bands of nicked and linearized pACY and pUCY vanish on the agarose gel after 30 min when treated with the Ala‐containing Cu(II)‐peptide **2**, which indicates that the reaction proceeded with cutting the plasmids (Figure 3e).

In comparison, incubating only Cu(II) or Asc with a plasmid (e.g. pUC19), as well as only adding peptide with Cu(II) or peptide with Asc, did not result in any linearization of the plasmid (Figure S17). Only in the presence of all components of the system, namely peptide, plasmid, Cu(II), and Asc, DNA linearization was observed. Notably, Cu(II) and Asc together result in weakly linearized and nicked DNA, which has been reported earlier in the literature [11].

**FIGURE 4:**
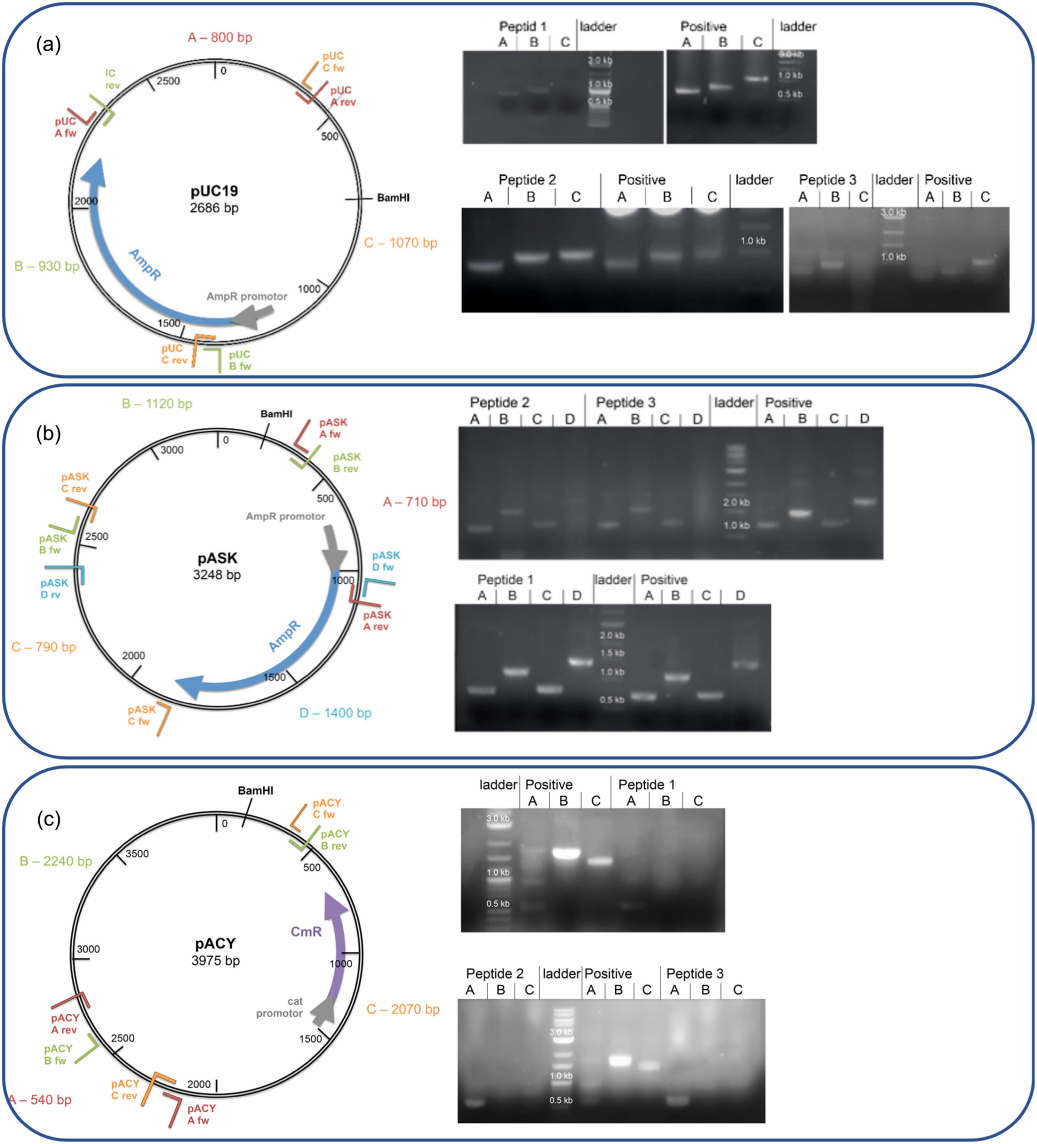
Plasmid map indicating cleavage regions A, B, C, and D and agarose gels showing the results from PCR of pUC19 (a) pASK (b), and pACY (c) after cleavage experiments with Cu‐peptide 1, Cu‐peptide 2, and Cu‐peptide 3, including positive controls using the plasmid only.

To gain insights into the mechanism and scission selectivity of the Cu‐peptides, we designed a set of DNA cleavage experiments that would allow the identification of preferred cleavage regions within the plasmids. Therefore, for each plasmid, three (pUC19 and pUCY) or four (pASK) regions—named A, B, C, and D—were defined to generate corresponding forward and reverse primers (Figure 4 and Figure S21) to be able to amplify and detect the DNA fragments of the respective regions via gel electrophoresis. As the plasmids were cut by all three Cu(II)‐peptides, the resulting linearized bands were cut out from the agarose gel, purified, and amplified through PCR with the corresponding primers. If the plasmid is preferably cut in one of those regions, it will no longer be recognized by the primers, thus not being amplified and not visible on the gel compared to a control experiment. The region C in pUC19 and region D of the pASK plasmid contain the antibiotic‐resistance‐introducing‐region, including the beginning of the sequence for AmpR. The plasmid pACY possesses antibiotic resistance against chloramphenicol.

To gain a deeper understanding of the scission results, we analyzed the nucleotide content of the regions where peptides were identified to preferably cut the DNA plasmids. Since AT‐rich sites were reported to be preferred for scission activity from experiments using short dsDNA (dodecanucleotides) [4], we analyzed the occurrence of the AATT, TTAA, ATAT, and TATA sites within the DNA sequences of the three plasmids (Table S1). For pUC19, we found that region C was not amplified when His‐containing peptide **1** and Asp‐containing peptide **3** in complex with Cu(II) were used for DNA cleavage (Figure 4a). This indicates that the plasmid was cut preferably within region C. In contrast, using Cu(II)‐peptide **2** (Ala‐containing) region C was still mostly intact as indicated by its corresponding gel band (Figure 4a). The region C of pUC19 contains around 60% of AATT and 75% of ATAT sites (Table S1). These results indicate that for peptide **1** and peptide **3**, the preferred scission region lies at AT‐rich regions, similar to other DNA‐binding small molecules and Arg‐Gly‐His tripeptide [4].

For pASK, results differ from those obtained for pUC19 (Figure 4a,b), as Cu(II)‐Ala‐ and Asp‐containing peptides **2** and **3** appear to cut the plasmid in the region D, while Cu(II)‐His‐containing peptide **1** does not show a preferred region (Figure 4b). The region D contains around 46% of AATT, which is more than in A, B, and C. However, it has only 33% of ATAT sites, whereas the greatest number of these sites is in region A, with around 50%.

Plasmid pACY emerged as more difficult for analysis. The PCR set‐up had to be altered to readily amplify the three fragments, A, B, and C (Figure 4c). The extension time was prolonged to 90 s and the annealing temperature to 56°C. Thus far, our results indicate that linearization is preferred within regions B and C as region A is visible in the agarose gel of all three peptides (Figure 4c). Thus, all three peptides possess preferred scission sites in AT‐rich regions B and C in pACY. Although pACY possesses antibiotic resistance against chloramphenicol and not ampicillin, like pUC19 and pASK, all three peptides showed the same preference to the resistance region of pACY alongside regions B and C.

Our results demonstrate that the Cu‐coordination environment has an impact on the scission ability of peptide‐Cu(II) complexes. Scission occurs even for the Cu(II) complex of an Ala‐containing peptide, which has no charged side chain, but a nonpolar Ala residue at the coordination site. These results are in agreement with the previously suggested cleavage mechanism where the metal ion coordination is crucial in a site‐specific manner, where ROS‐induced damage of the DNA strand occurs [35].

### 3.3 Peptide‐DNA Binding Studies

To further investigate peptide‐DNA scission, we examined the binding of peptides to plasmids using the displacement of two fluorescent dyes, Hoechst 33 258 and ethidium bromide. A reduction in fluorescence indicated dye displacement by the peptides from DNA. We compared the three metallopeptides to the classic Gly‐Gly‐His ATCUN peptide (referred to as the GGH‐peptide, Figure S4).

Hoechst 33 258 (bisBenzimide H 33 258) binds DNA in two distinct modes [25]. Minor groove binding, which preferentially targets AT‐rich regions, yielding a high quantum yield and an emission maximum around 460 nm. This binding mode dominates at dye‐to‐DNA phosphate molar ratios below 0.05 [25, 36].

**FIGURE 5:**
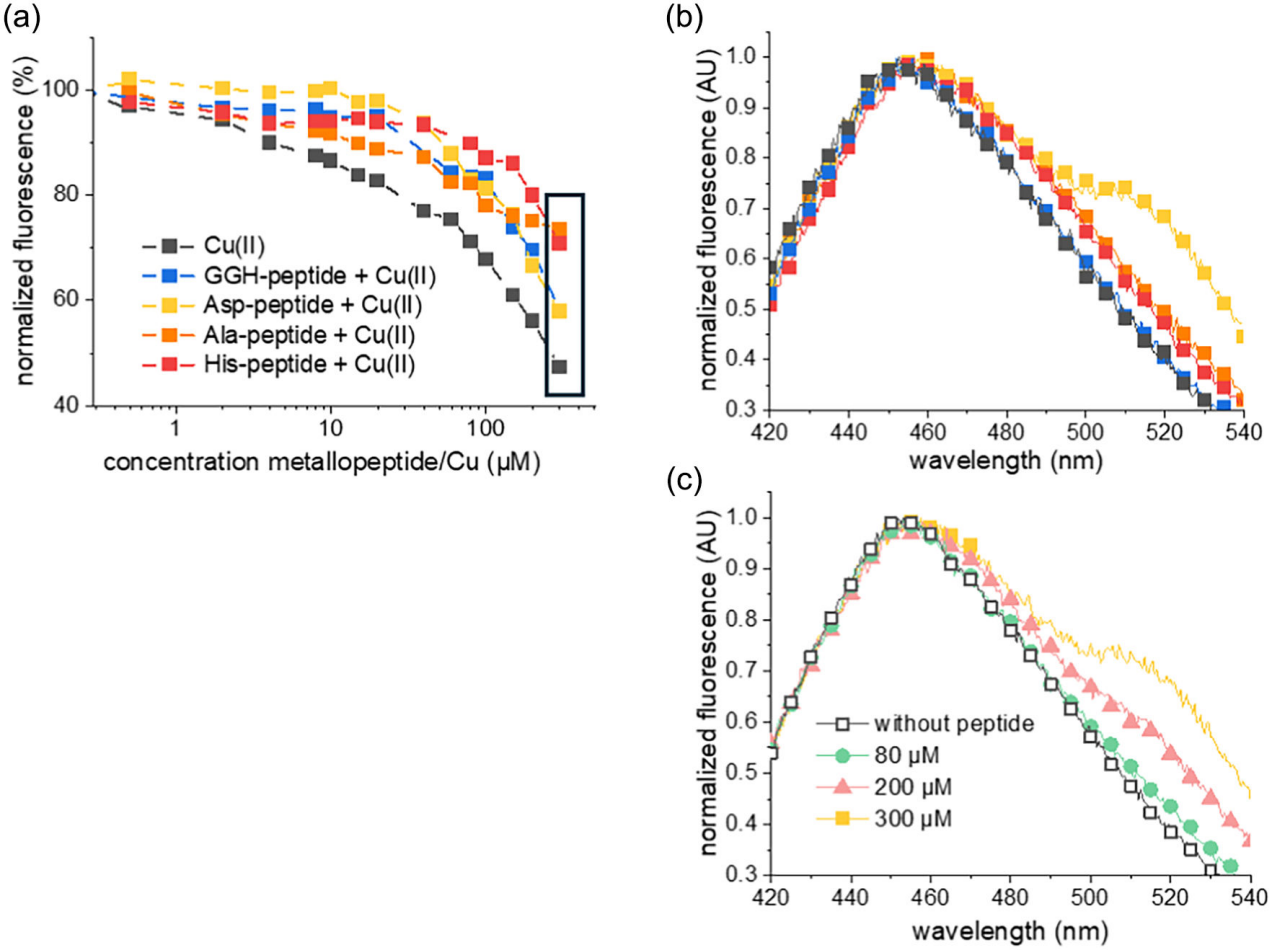
Displacement of Hoechst 33 258 from the pASK plasmid by titration with ATCUN metallopeptides and Cu(II) alone. (a) Emission at the 455 nm peak, normalized to 100% on fluorescence in the absence of the peptides and Cu(II). The values were corrected for the added volume of metallopeptide solution. (b) Normalized fluorescence, concentration of metallopeptides and Cu(II) is 300 µM (in (a), the corresponding values are enframed). (c) Normalized fluorescence in the presence of Asp‐metallopeptide at concentrations from 0 to 300 µM.

For all plasmids, Cu(II) alone, in the absence of peptides, significantly displaces the dye (Figure 5, Figure S22), and the peptides do not enhance the displacement, similarly to the classic GGH‐ATCUN (Figure S23). High concentrations of peptides, above 30 µM, cause a redshift of the Hoechst fluorescence peak from 460 to ~490 nm, but this shift also occurs in the absence of DNA (Figure S22).

Ethidium bromide, an intercalating dye, was similarly studied. Previous work has demonstrated its displacement by ATCUN peptides on a dodecanucleotide [37, 38]. For the larger plasmids used here, the displacement of ethidium bromide by all metallopeptides closely resembles that caused by Cu(II) alone (Figure S24). This suggests that the metallopeptides do not exhibit additional binding to ethidium bromide intercalation sites at the plasmid scale.

## 4 Conclusion

ATCUN‐like peptides containing a histidine (His) residue in their sequences have been extensively studied for DNA cleavage. In this study, we investigated the ability of His‐free ATCUN‐like motifs to linearize DNA. All three peptides demonstrated similar binding to Cu(II), indicating that His is not essential for the formation of Cu(II)‐peptide complexes. Further, the use of Tris buffer does not prevent the complex formation. However, the Tris‐metal binding may increase the molar ratios at which metallopeptides are formed.

Peptides containing Ala or Asp in place of His were capable of linearizing the three plasmids studied. Specific cleavage region analysis showed that the Asp‐containing peptide exhibited the same site specificity as the His‐containing peptide, preferentially cleaving at AATT‐ and ATAT‐rich sites. In contrast, the Ala‐containing peptide displayed different behavior, cleaving two plasmids (pUC19 and pASK) without regional specificity, while still preferring AATT and ATAT regions in the pACY plasmid.

These findings demonstrate that His is not strictly necessary for DNA linearization by ATCUN‐like peptides and that some peptides exhibit sequence‐specific cleavage preferences. This insight holds significant potential for rational design of small metallopeptides for applications ranging from anticancer therapies [39] to antimicrobial treatments [40].

## Funding

This study was supported by Liebig Fellowship, iNAPO, Knut and Alice Wallenberg Foundation, Vetenskapsrådet (2025‐05306), Cancerfonden (254861).

## Conflicts of Interest

The authors declare no conflicts of interest.

## Supporting information

Supplementary Material

## Data Availability

The data that supports the findings of this study are available in the supplementary material of this article.
