# Supplementary material for: DNA Scission by Non‐Histidine Amino‐Terminal Cu(II) and Ni(II) Binding‐Like Peptides
Source: Chembiochem. 2026 May 25;27(10):e70397. doi: 10.1002/cbic.70397 (PMC13206376; doi:10.1002/cbic.70397)

Supporting Information

DNA Scission by Non-Histidine Amino-Terminal Cu(II) and Ni(II) Binding-Like Peptides

Lena K. Müller,[†1] Hanna Zhdanova,[†3] Ivan N. Unksov,[†3] Alexander Gräwe,[2] Joshua Stahl,[1] Viktor Stein[2], Daniel Tietze,[3] and Alesia A. Tietze[3]*

[1] Clemens Schöpf Institute for Organic Chemistry and Biochemistry, Darmstadt University of Technology, Alarich-Weiss-Str. 4, 64287 Darmstadt, Germany
[2] Darmstadt University of Technology, Department of Biology, Schnittsphanstr.12, 64287 Darmstadt, Germany
[3] Department of Chemistry and Molecular Biology, Wallenberg Centre for Molecular and Translational Medicine, University of Gothenburg, Natrium, Medicinaregatan 7B, 41390 Gothenburg, Sweden,
Email: alesia.a.tietze@gu.se

[†] authors contributed equally to this work

Content

Analytical data 2
DNA scission data 9
Fluorescence displacement data 12

Analytical data

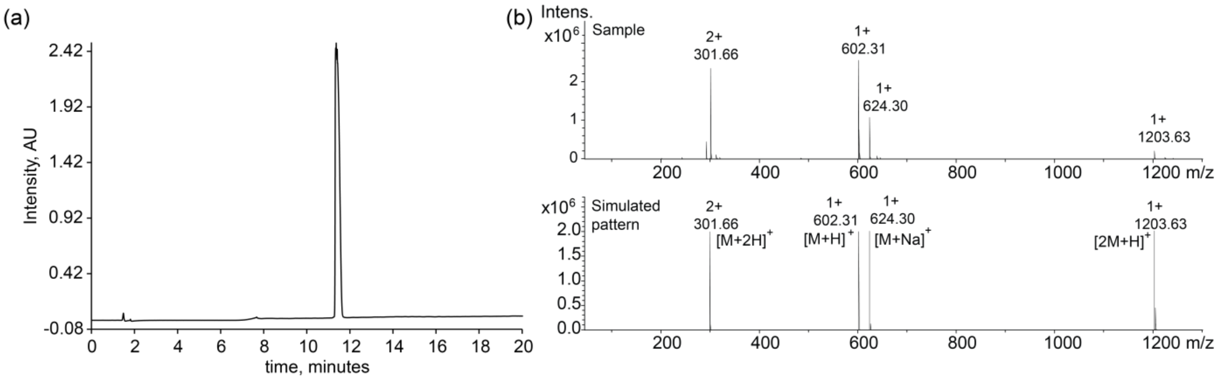

Figure S1 | RP-HPLC and inserted ESI-MS of purified His-containing peptide 1. HPLC gradient: 0% for 4 minutes followed by a gradient of 0 – 30% eluent B in 20 minutes, detection at 214 nm.

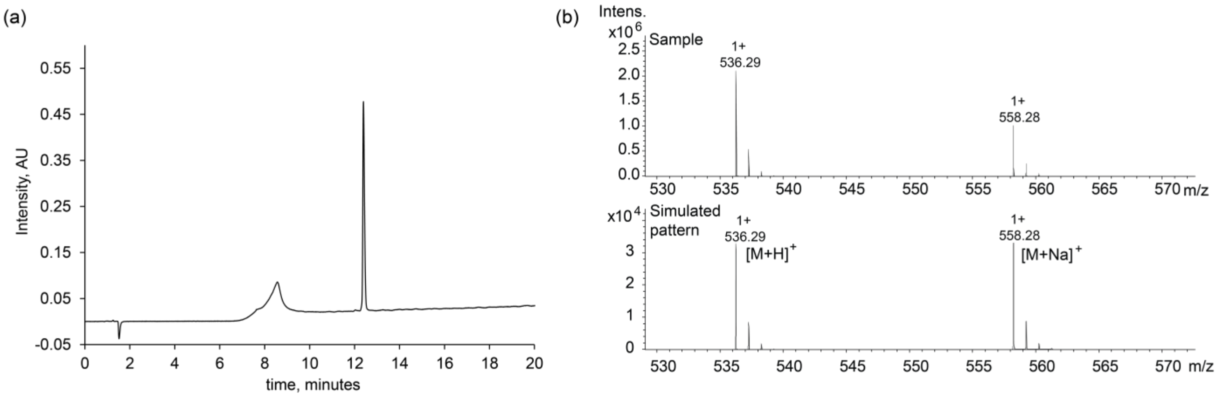

Figure S2. RP-HPLC and inserted ESI-MS of purified Ala-containing peptide 2. HPLC gradient: 5% for 4 minutes followed by a gradient of 5 – 30% eluent B in 20 minutes total, detection at 214 nm. Peak at 8 min refers to the column impurity.

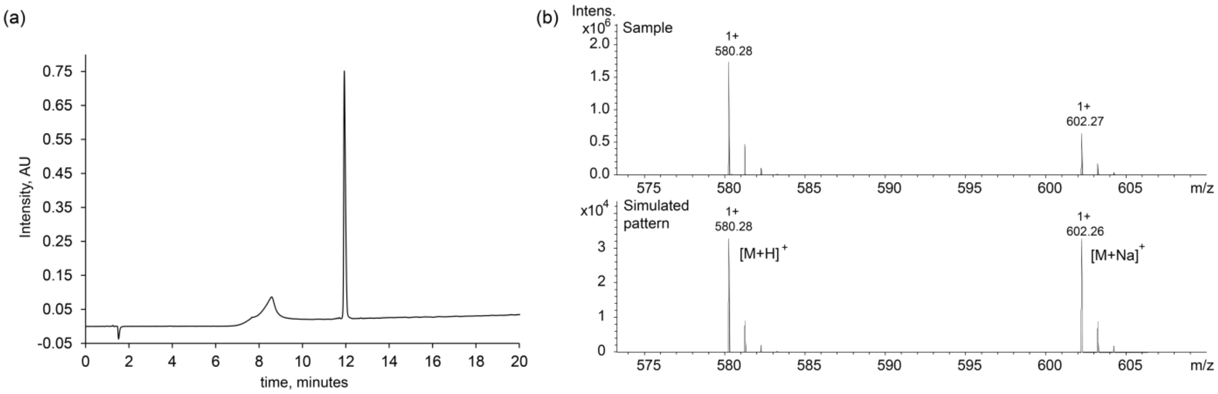

Figure S3. RP-HPLC and inserted ESI-MS of purified Asp-containing peptide 3. HPLC gradient: 5% for 4 minutes followed by a gradient of 5 – 30% eluent B in 20 minutes total, detection at 214 nm. Peak at 8 min refers to the column impurity.

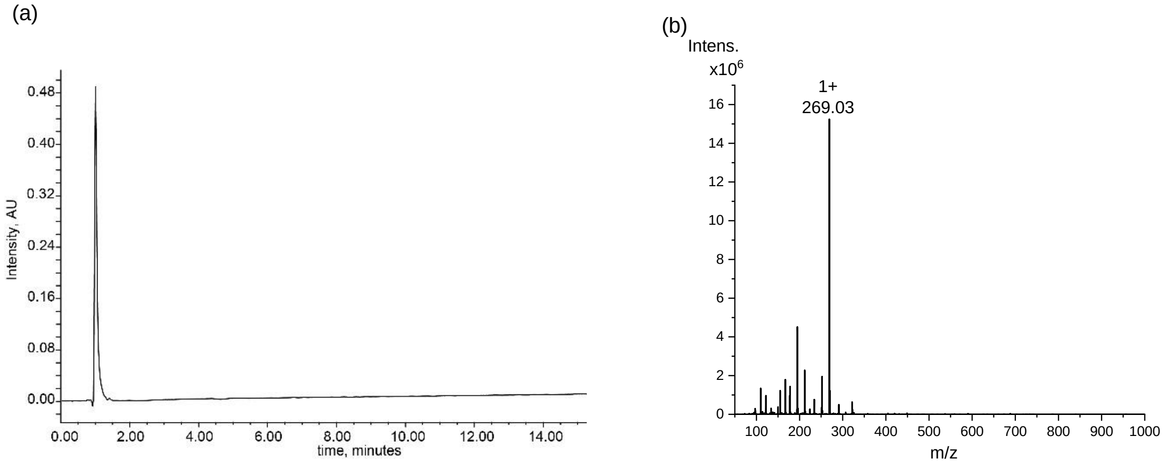

Figure S4. RP-HPLC and ESI-MS of purified GGH ATCUN peptide. HPLC gradient: 0 – 15% eluent B in 15 minutes total.

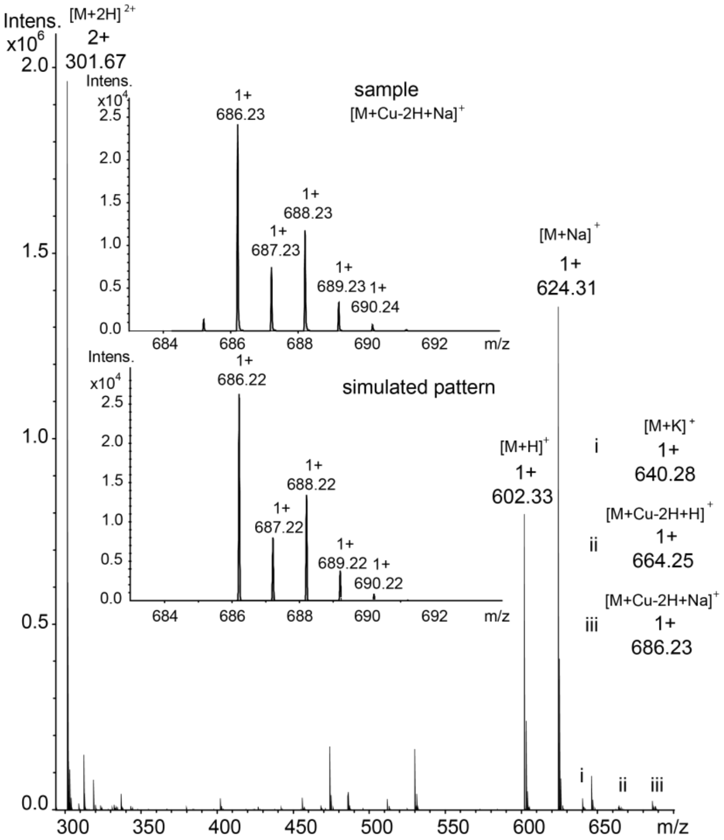

Figure S5. His-containing peptide 1 - coordination Cu(II) in Phosphate buffer pH 7.4

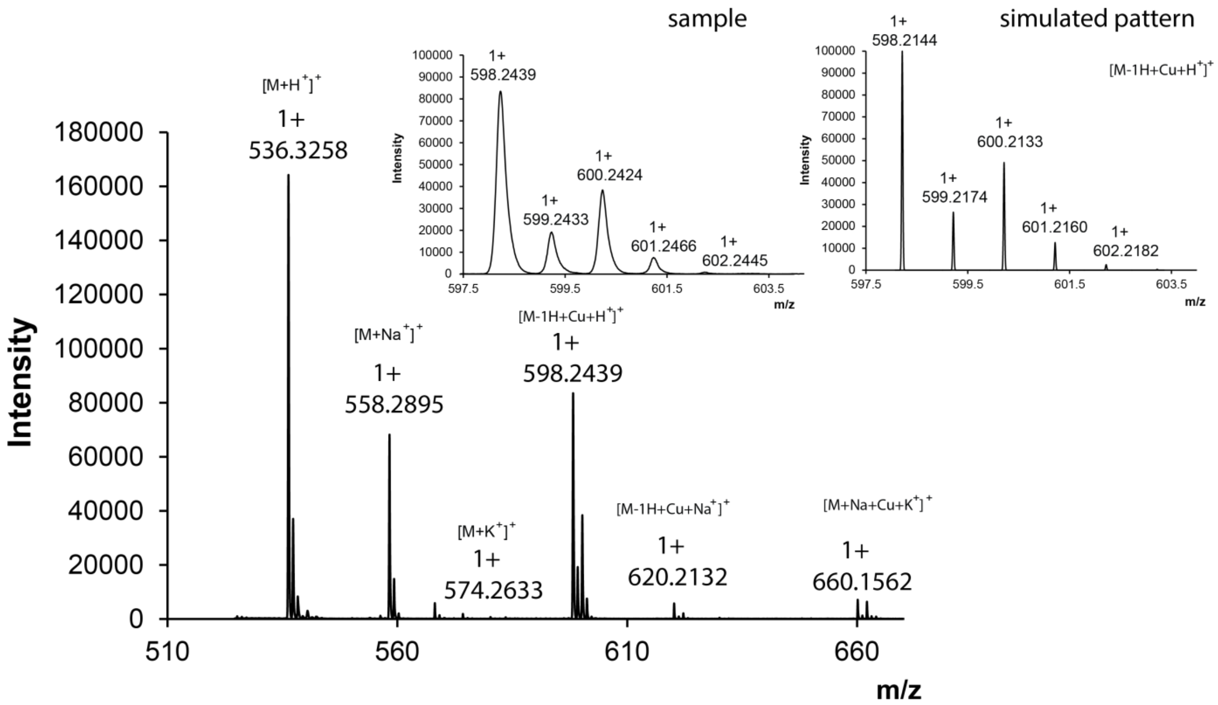

Figure S6. Ala-containing peptide 2 - coordination Cu(II) in Phosphate buffer pH 7.4

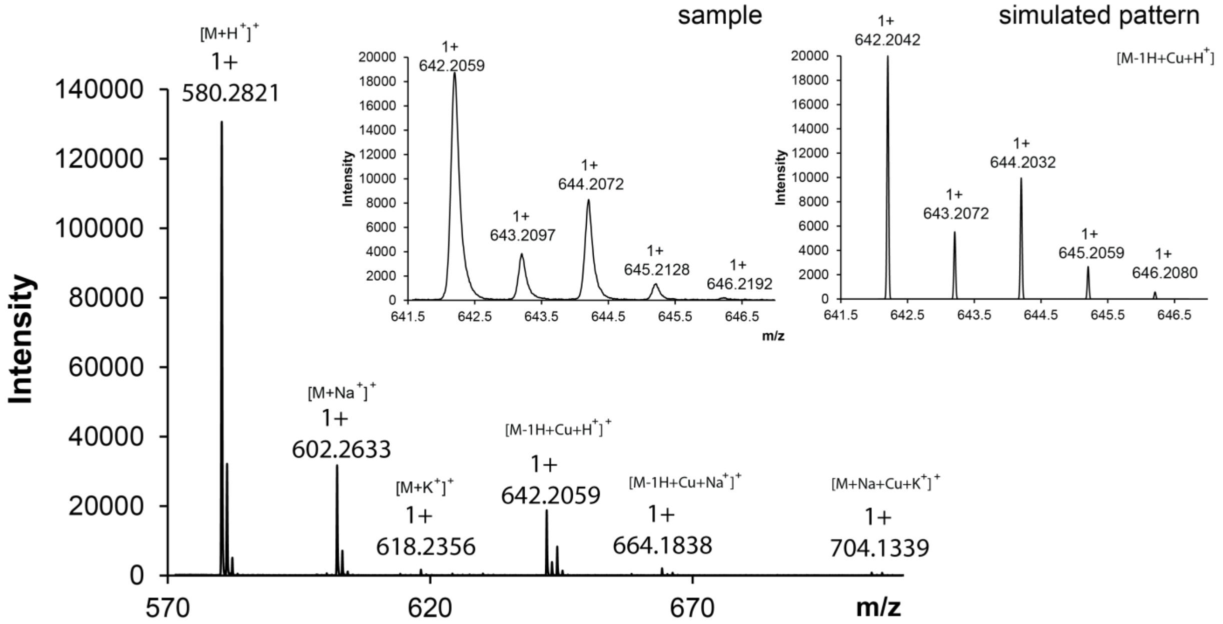

Figure **S7**. Asp-containing peptide 3 - coordination Cu(II) in Phosphate buffer pH 7.4

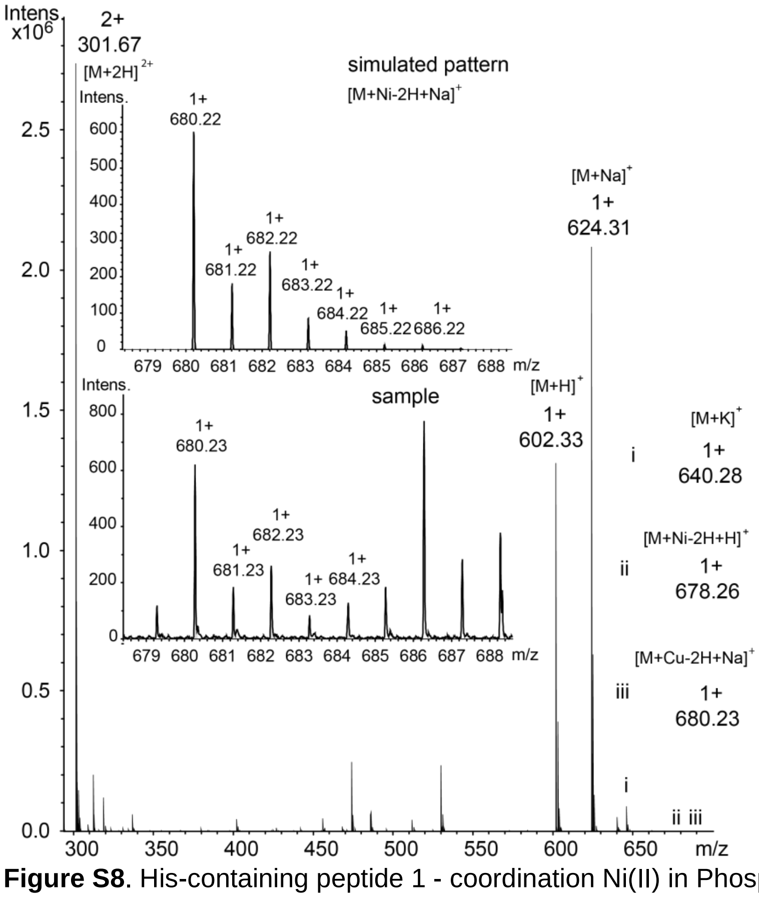

Figure S8. His-containing peptide 1 - coordination Ni(II) in Phosphate buffer pH 7.4

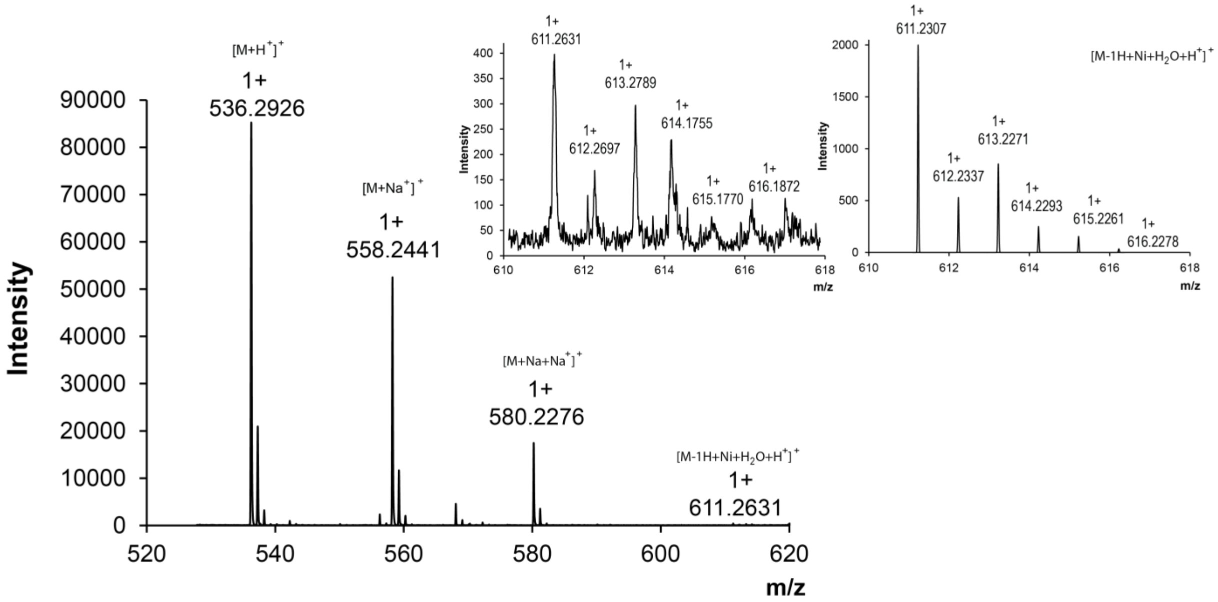

Figure S9. Ala-containing peptide 2 - coordination Ni(II) in Phosphate buffer pH 7.4

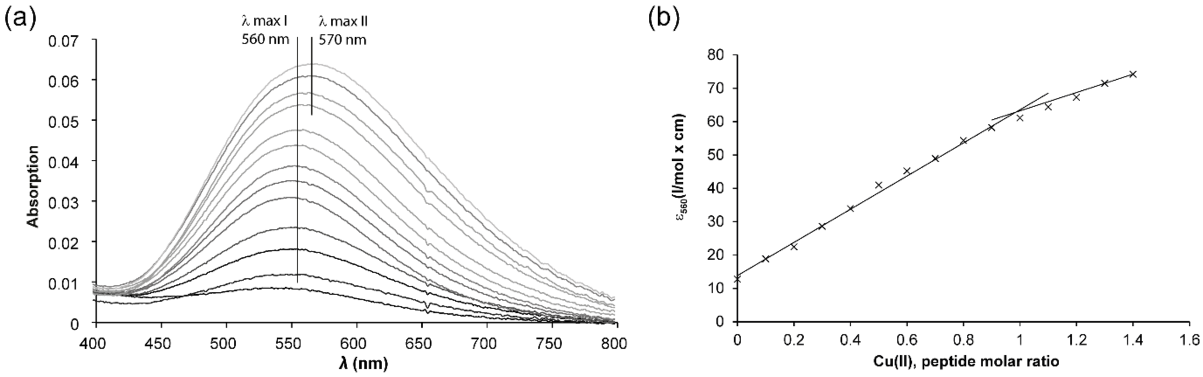

Figure S10. **UV-Vis titration of peptide 1 and Cu(II)**. Peptide 1 is dissolved to yield a 1 mM solution in Tris buffer pH 8.0 and 0.1 equivalents of Cu(II) added from a $CuSO_4$ solution in water. (a) UV-Vis spectral region characteristic for metal binding. The vertical lines visualize the absorption maximum below 1 eq. (λ max I) and when metal addition exceeded 1 eq. M(II) (λ max II). (b) increase of extinction coefficient with addition of Cu(II).

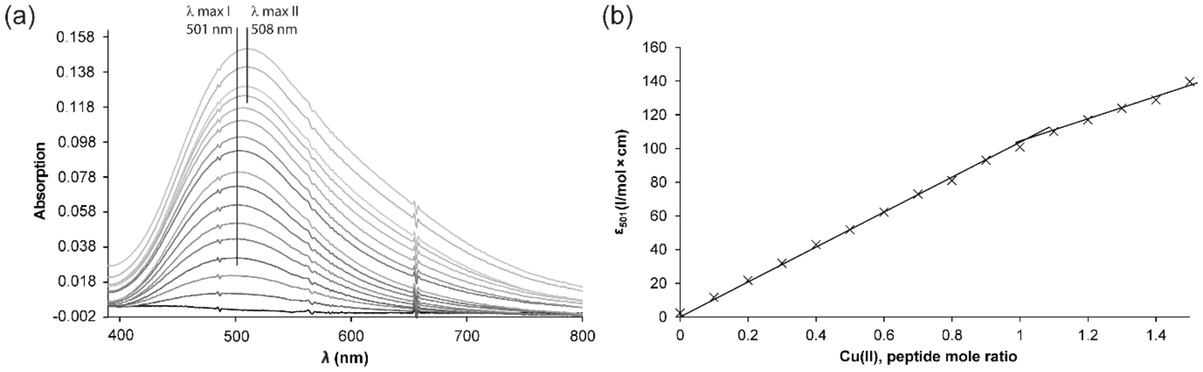

Figure S11. UV-Vis titration of peptide 2 and Cu(II). Peptide 2 is dissolved to yield a 1 mM solution in Tris buffer pH 10.5 and 0.1 equivalents of Cu(II) added from a $CuSO_4$ solution in water. (a) UV-Vis spectral region characteristic for metal binding. The vertical lines visualize the absorption maximum below 1 eq. (λ max I) and when metal addition exceeded 1 eq. M(II) (λ max II). (b) increase of extinction coefficient with addition of Cu(II).

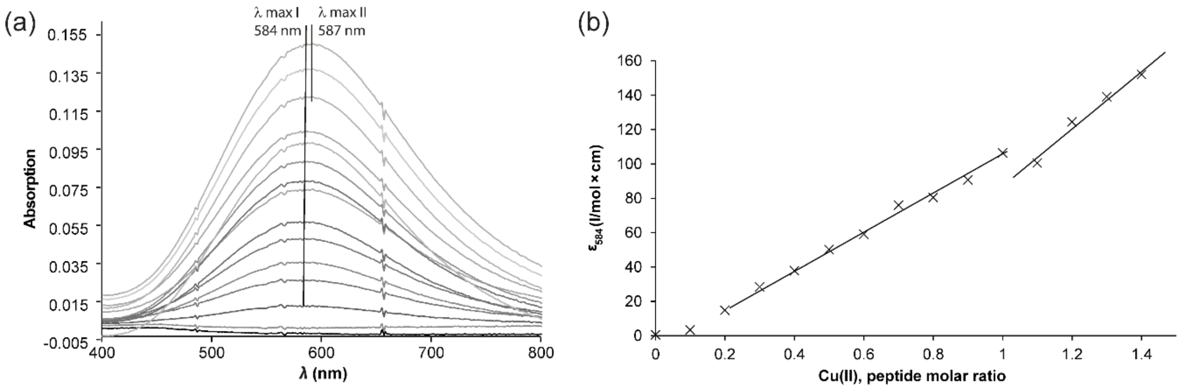

Figure S12. UV-Vis titration of peptide 3 and Cu(II). Peptide 3 is dissolved to yield a 1 mM solution in Tris buffer pH 8.0 and 0.1 equivalents of Cu(II) added from a $CuSO_4$ solution in water. (a) UV-Vis spectral region characteristic for metal binding. The vertical lines visualize the absorption maximum below 1 eq. (λ max I) and when metal addition exceeded 1 eq. M(II) (λ max II). (b) increase of extinction coefficient with addition of Cu(II).

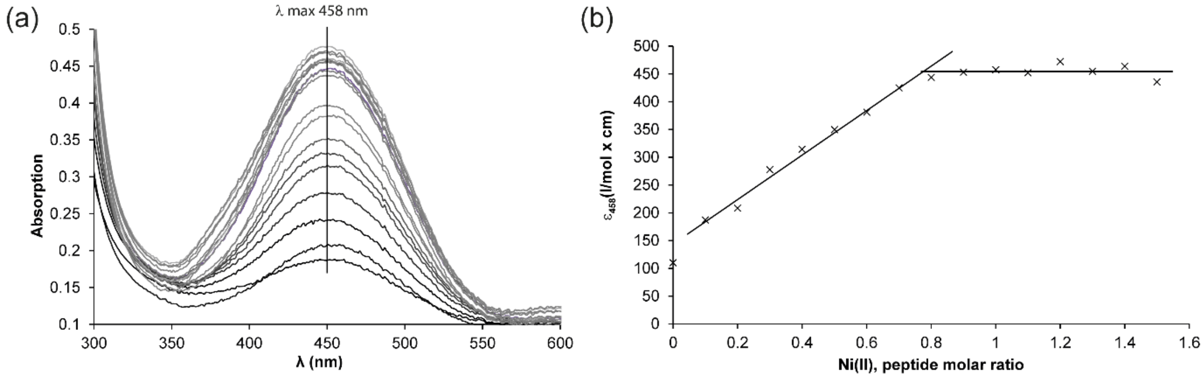

Figure S13. **UV-Vis titration of peptide 1 and Ni(II)**. Peptide 1 is dissolved to yield a 1 mM solution in Tris buffer pH 10.5 and 0.1 equivalents of Ni(II) added from a $NiSO_4$ solution in water. (a) UV-Vis spectral region characteristic for metal binding. The vertical lines visualize the absorption maximum. (b) Increase of extinction coefficient with addition of Ni(II).

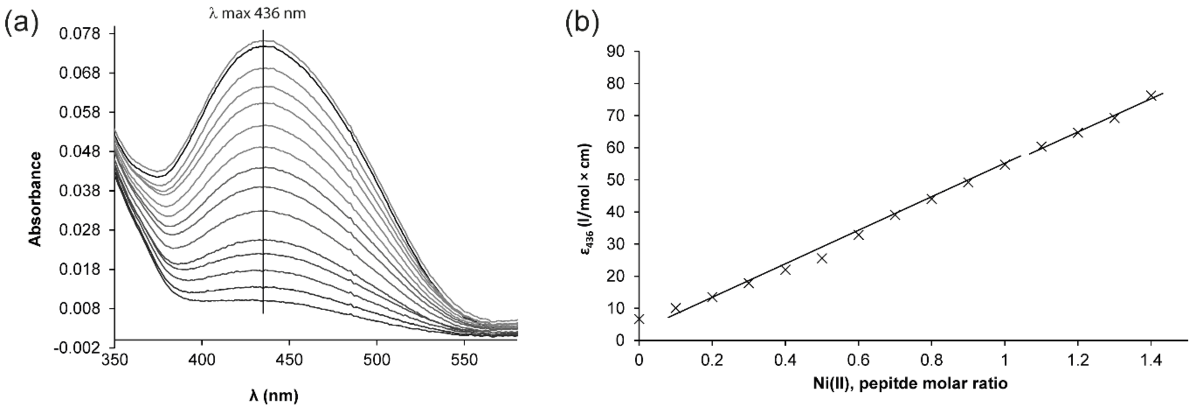

Figure S14. UV-Vis titration of peptide 2 and Ni(II). Peptide 2 is dissolved to yield a 1 mM solution in Tris buffer pH 10.5 and 0.1 equivalents of Ni(II) added from a $NiSO_4$ solution in water. (a) UV-Vis spectral region characteristic for metal binding. The vertical lines visualize the absorption maximum. (b) Increase of extinction coefficient with addition of Ni(II).

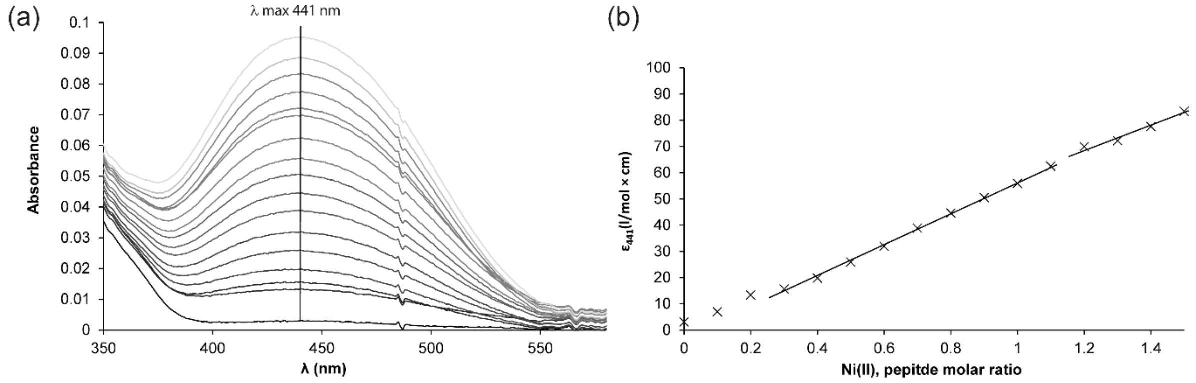

Figure S15. UV-Vis titration of peptide 3 and Ni(II). Peptide 3 is dissolved to yield a 1 mM solution in Tris buffer pH 10.5 and 0.1 equivalents of Ni(II) added from a $NiSO_4$ solution in water. (a) UV-Vis spectral region characteristic for metal binding. The vertical lines visualize the absorption maximum. (b) Increase of extinction coefficient with addition of Ni(II).

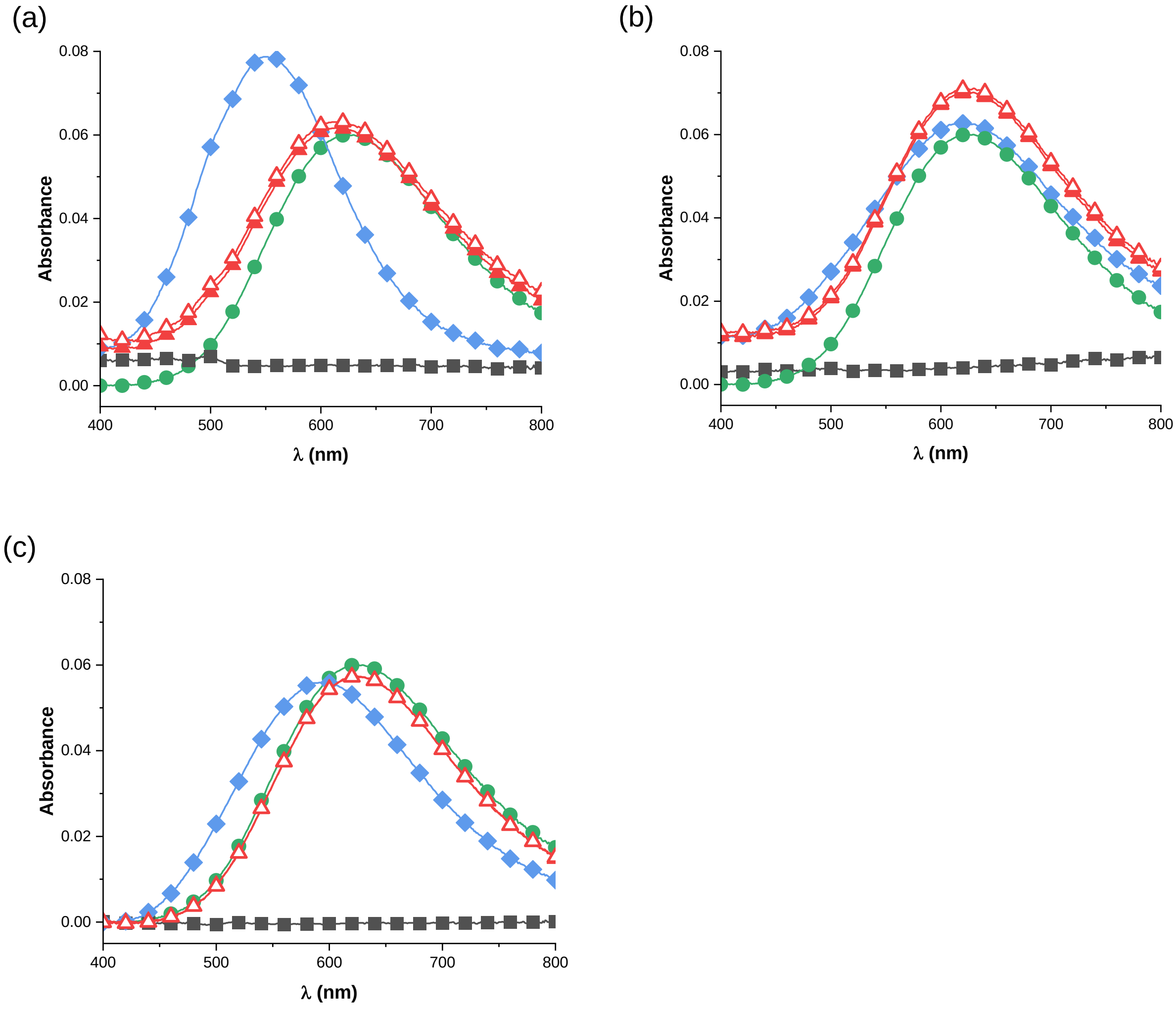

Figure S16. UV-Vis spectra of peptides 1-3 and glutathione in complexes with Cu(II). Spectra for peptides 1-3 (**panels a-c respectively**) were firstly recorded at 2 mM without Cu(II) (gray square markers), then in presence of Cu (II) at 1 mM (0.5 equivalents, blue diamond markers), after which glutathione was added (the spectra immediately after addition are depicted in filled red triangle markers, and 1 hour after addition – in open red markers) to a final concentration of 3 mM to compete with the peptides. For reference, we used absorption of a similar glutathione + Cu(II) solution without the peptides 1-3 (green circle markers). All solutions were in Tris buffer pH 8.0. Added volumes of Cu(II) and glutathione solutions were small compared to initial sample volumes and did not cause absorbance losses due to dilution.

DNA scission data

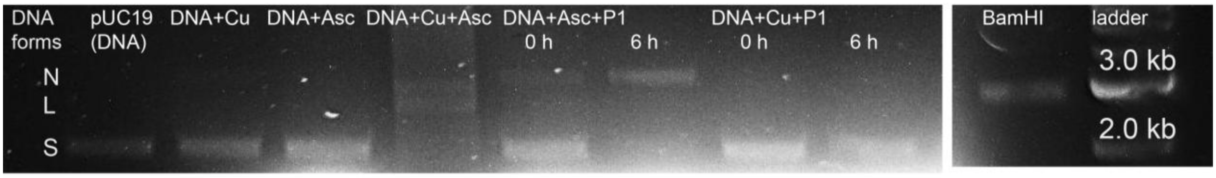

Figure S17. DNA conformation as affected by control reagents. Cu(II) without peptides, ascorbate (Asc) without peptides, Cu(II) + ascorbate, Cu(II) + peptide 1.

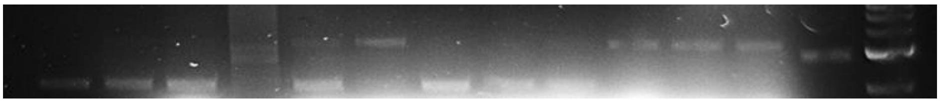

Figure S18. Original uncut agarose gel from Figure S16.

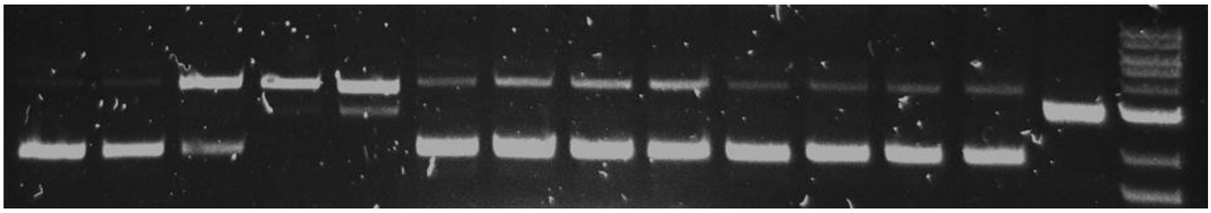

Figure S19. Original uncut agarose gel for scission of the pUC19 plasmid using peptide 1 (100 µM) + Cu(II) (90 µM) or Ni(II) (90 µM) + ascorbate (Asc, 100 µM). Cut gel is in the Figure 3.

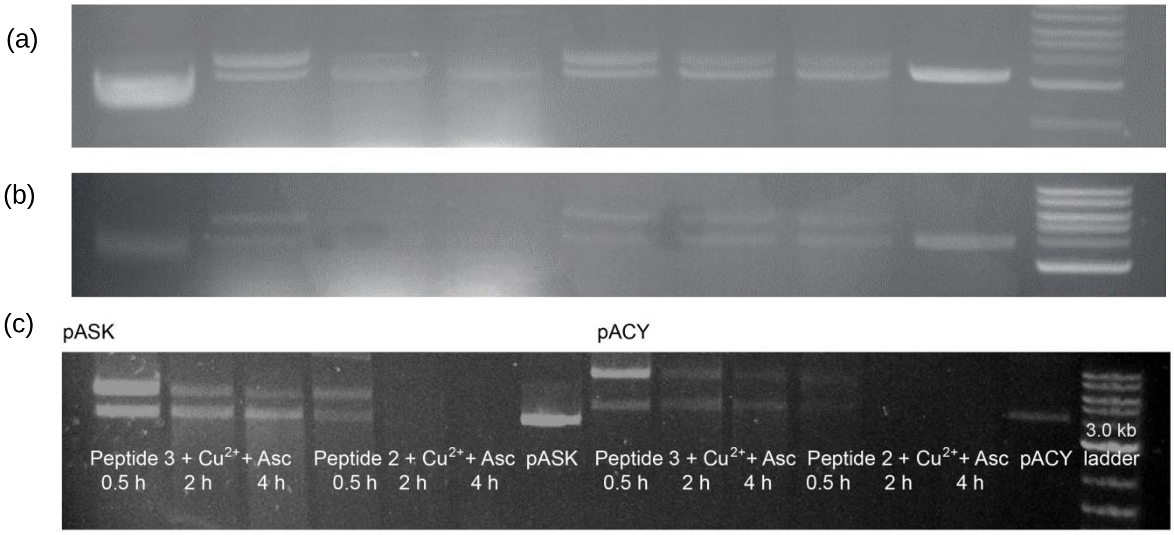

Figure S20. Original uncut agarose gels showing scission of **(a)** pASK by peptide 1 + Cu(II) + Asc; **(b)** pACY by the same; **(c)** pASK and pACY by peptide 2 + Cu^{2+} + Asc and peptide 3 + Cu^{2+} + Asc. Cut gels are in the Figure 3.

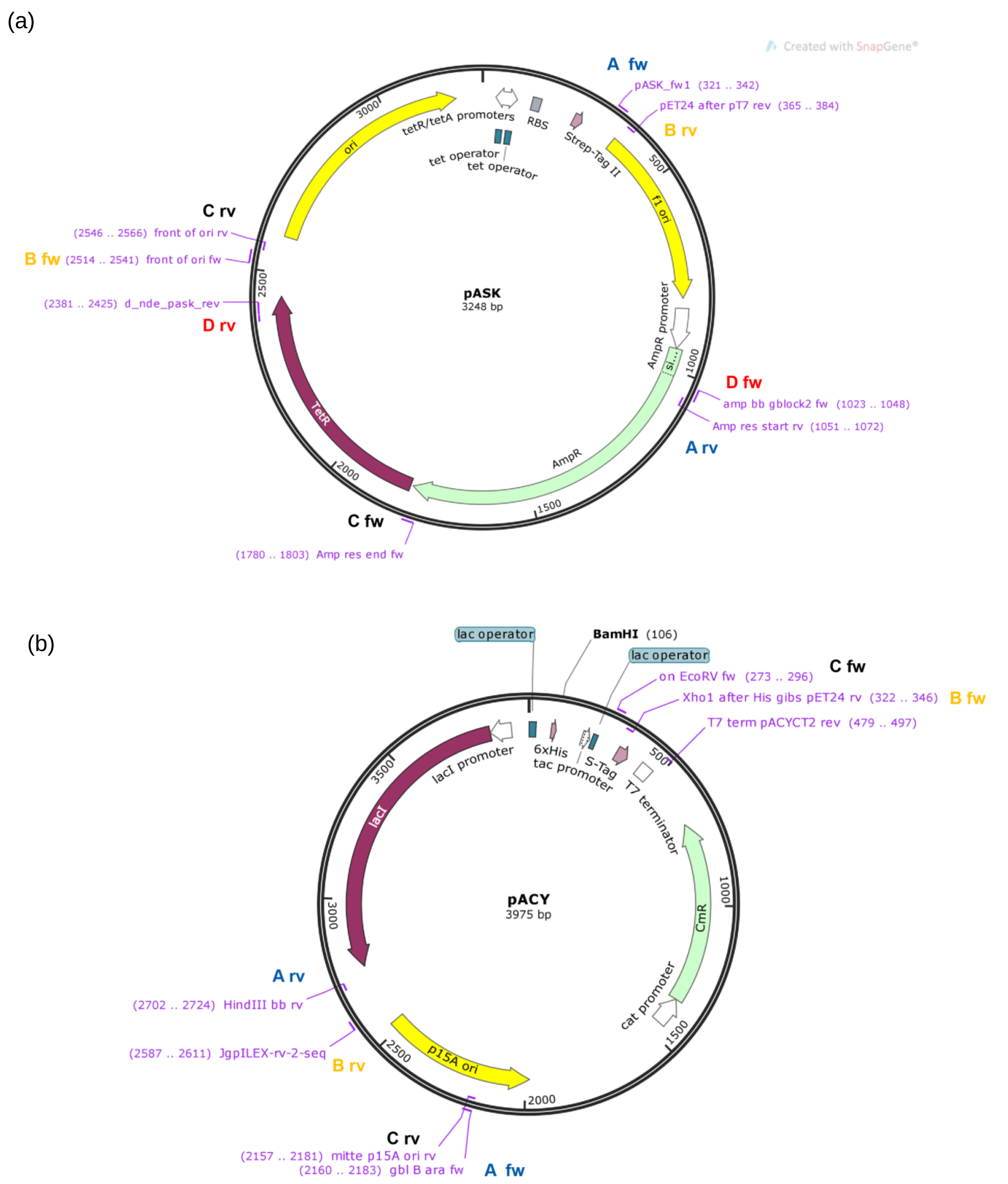

Figure S21. (a) Map of pASK showing only the used primers (reversed rv and forward fw) and the restriction enzyme BamHI; **(b)** map of pACY showing only the used primers (reversed rv and forward fw) and the restriction enzyme BamHI

Table S1. occurrence of the AATT/TTAA motif within the sequence of the plasmids studied in this work

		region A	region B	region C	region D	total N° of repetitions found
pUC19	AATT	0/0%	3/43%	4/57%	-	7
	TTAA	1/8%	8/62%	5/38%	-	13
	ATAT	0/0%	1/25%	3/75%	-	4
	TATA	1/33%	1/33%	1/33%	-	3
pASK	AATT	3/23%	4/31%	4/31%	6/46%	13
	TTAA	7/33%	5/24%	9/43%	8/38%	21
	ATAT	3/50%	1/16.7%	2/33.3%	2/33%	6

	TATA	1/25%	2/50%	1/25%	1/25%	4
pACY	AATT	3/13%	7/30%	16/70%	-	23
	TTAA	2/9%	9/39%	13/57%	-	23
	ATAT	2/11%	11/61%	7/39	-	18
	TATA	1/7%	8/57%	6/43%	-	14

Fluorescence displacement data

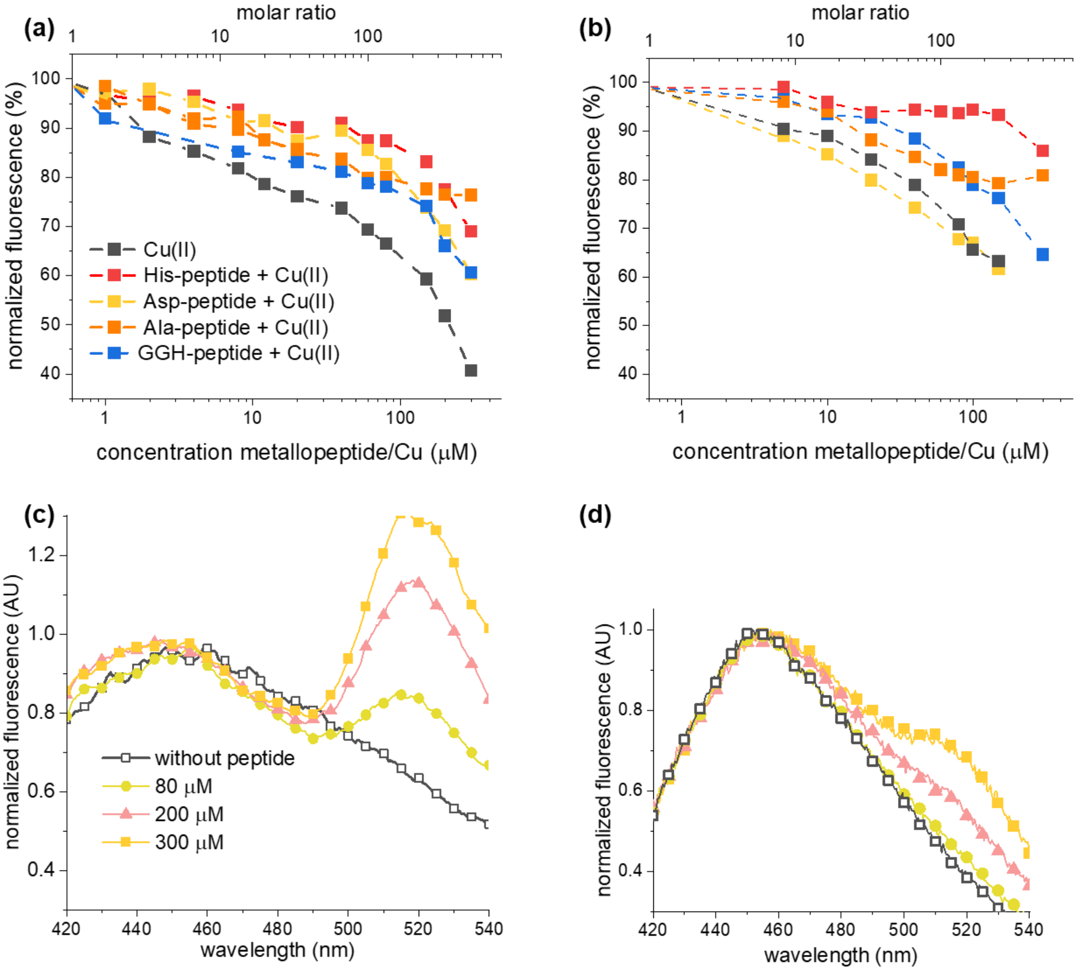

Figure S22. Displacement of Hoechst 33258 from the (a) pACY and (b) pUC19 plasmids by titration with metallopeptides and Cu(II) alone. (a, b) Emission at the 455 nm peak, normalized to 100% on fluorescence in absence of the peptides and Cu(II). The values were corrected for the added volume. (c) Normalized fluorescence of Hoechst in absence and in presence of Asp-metallopeptide at high concentrations. No plasmid is present. (d) Normalized fluorescence of Hoechst bound to the pACY plasmid in absence and in presence of Asp-metallopeptide at high concentrations.

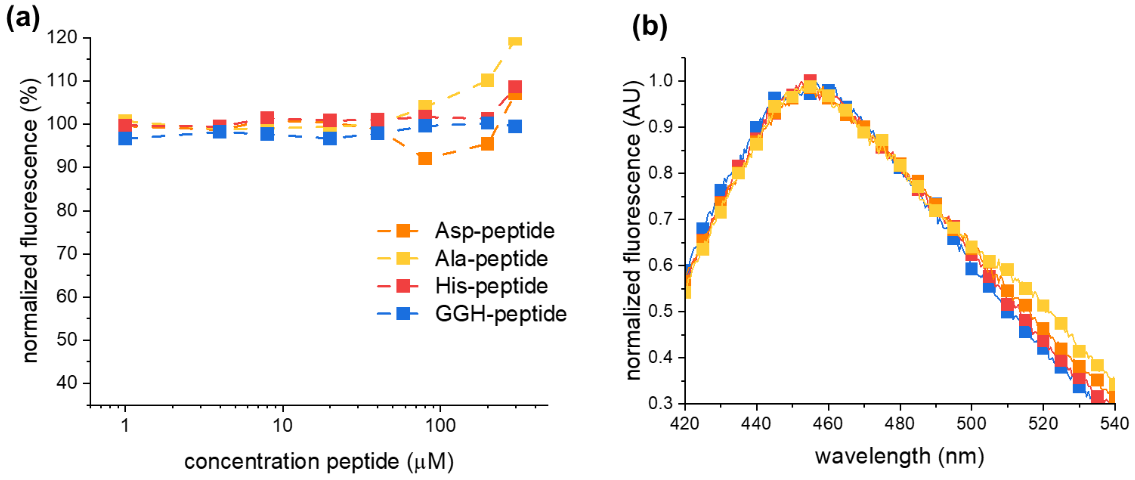

Figure S23. Displacement of Hoechst 33258 from the pASK plasmid by titration with peptides without Cu(II). (a, b) Emission at the 455 nm peak, normalized to 100% on fluorescence in absence of the peptides. The values were corrected for the added volume. (b) Normalized fluorescence, concentration of peptides is 300 µM.

Figure S24. Displacement of ethidium bromide from the pUC19 plasmid by titration with metallopeptides and Cu(II) alone. Emission was measured at the 595 nm peak and normalized to 100 % on fluorescence in absence of the peptides and Cu(II), the values were corrected for the added volume.

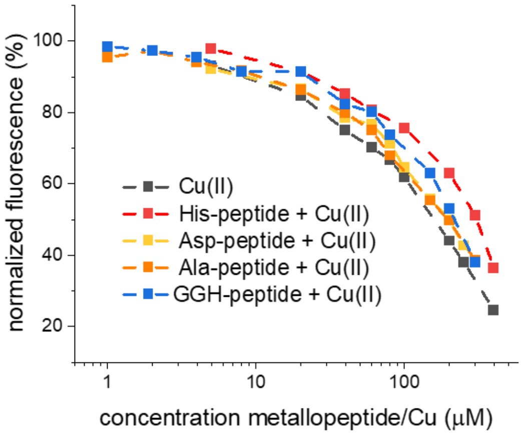

Supplement: Supplementary file 1 — Supplementary Material [file CBIC-27-e70397-s001.pdf]
